# Efficacy of brachytherapy for locally advanced bladder cancer: a single-center retrospective clinical study

**DOI:** 10.1080/15384047.2025.2509200

**Published:** 2025-05-22

**Authors:** Xuebing Han, Huiqing Chen, Bin Wang

**Affiliations:** Department of Urology, Shanxi Province Cancer Hospital/Shanxi Hospital Affiliated to Cancer Hospital, Chinese Academy of Medical Sciences/Cancer Hospital Affiliated to Shanxi Medical University, Taiyuan, China

**Keywords:** Bladder cancer, brachytherapy, radiotherapy, radioactive particles

## Abstract

To explore the feasibility, safety, and effectiveness of brachytherapy of locally advanced bladder cancer, clinical data of 86 patients with locally advanced bladder cancer treated in the Department of Urology Surgery, Shanxi Provincial Cancer Hospital, between January 2015 and June 2019 were analyzed retrospectively. The patients were categorized into the study (*n* = 45) and control (*n* = 41) groups according to the treatment methods. Patients in the study group were treated with brachytherapy (intraoperative implantation of radioactive particles) + neoadjuvant chemotherapy (NAC), and those in the control group were treated with NAC. Patients in both groups underwent radical cystectomy (RC) + pelvic lymph node dissection. Postoperative pathological examinations proved that patients in both groups had urothelial carcinoma at stage pT_3_–pT_4_. The endpoints included 3-y locoregional recurrence-free survival (LRFS), distant metastasis-free survival (DMFS), disease-free survival (DFS), overall survival (OS), and adverse events after treatment. The efficacy and safety of interstitial implantation of radioactive particles for the treatment of locally advanced bladder cancer were assessed. The patients were followed up for 9–42 months. The 3-y LRFS was significantly higher in the study group (88.9%) than in the control group (60.9%) (*p* = .003). The 3-y DMFS in the study group (71.1%) and the control group (73.2%) was statistically similar (*p* = .945). The 3-y DFS and OS were not statistically significant between the two groups (DFS: study group 64.4% *vs*. control group 51.2%, *p* = .073; OS: study group 66.7% *vs*. control group 58.5%, *p* = .180). Local shifting of the particles was detected in three patients at 1 week to 1 month after the operations in the study group, but no related complications were observed. Blood events (anemia, leukocytopenia, and thrombocytopenia), liver and renal dysfunction, vomiting, diarrhea, and weakness were the major adverse reactions, which were alleviated after symptomatic treatments. The results have not statistically significant differences between the two groups in major adverse reactions. Compared to the NAC group, brachytherapy + NAC significantly prolongs the LRFS of patients with locally advanced urothelial bladder carcinoma who underwent RC + pelvic lymph node dissection. This surgery increases the LRFS, develops better personalized treatment plans, and improves treatment effectiveness. In addition, the treatment is safe and effective, with only limited adverse effects.

## Introduction

Urothelial carcinoma (UC) is one of the most common malignant tumors in the Urology Department, which includes upper tract urothelial carcinoma (UTUC) and urothelial bladder carcinoma (UBC). The incidence of UC is high, and the majority of the cases are UBC.^1^ The incidence of bladder cancer ranks 9^th^ among all malignant tumors globally; specifically, the incidence ranks 7^th^ in males and is <10^th^ in females. The mortality rate of bladder cancer ranks 13^th^ among all malignant tumors.^2^ According to the stages, primary bladder cancers can be classified into non-muscle-invasive bladder cancer (NMIBC) and muscle-invasive bladder cancer. Cisplatinum-based neoadjuvant chemotherapy (NAC), followed by radical cystectomy (RC) + pelvic lymph node dissection, has been the standard treatment for patients with MIBC for >10 y, which improves the survival rate of patients and prevents local recurrence and distal metastases.^3–5^ Recent studies have shown evolving treatment trends for muscle-invasive bladder cancer, such as in Germany from 2006 to 2019, reflecting advancements and changes in clinical practices.^6^ However, tumor recurrence at the surgical site in the pelvic cavity could occur in approximately 30% patients with locally advanced (T_3_-T_4_) UBC.^7–11^ Moreover, the local recurrence is associated with low cancer-specific survival,^12,13^ which exhibits poor effects of salvage treatment,^9,11–14^ and the median survival is only about 9 months.^9,15^ The clinical trials have shown that NAC reduces the risk of locoregional recurrence,^16^ significantly improves the disease-free survival (DFS),^17,18^ and thus might prolong the survival.

Comparing to conventional radiotherapy, brachytherapy (interstitial implantation of radioactive particles) has definite treatment efficacy and limited side effects in the treatment of solid tumors and hence has already been widely utilized. Hitherto, only a few studies are available on the treatment of UBC by brachytherapy. Therefore, the present study retrospectively analyzed the clinical data of 86 patients with locally advanced (stage T_3_-T_4_) UBC that underwent brachytherapy+ NAC or NAC. Also, the effectiveness and safety of the regimen of brachytherapy + NAC were assessed. This study is a part of a series of studies on the interstitial implantation of radioactive particles for the treatment of UC.^19^

## Materials and methods

### Clinical data

A total of 86 patients with locally advanced (stage T_3_-T_4_) UBC, according to the TNM staging criteria (8^th^ edition) issued by the Union for International Cancer Control (UICC), were treated in the Department of Urology Surgery, Shanxi Provincial Cancer Hospital between January 2015 and June 2019 were included. The patients were categorized according to the treatments into two groups, i.e. the study group that underwent brachytherapy + NAC and the control group that received only NAC. The Shanxi Provincial Cancer Hospital has been qualified for brachytherapy for the treatment of solid tumors (including UC) in 2001. The study was approved by the Ethics Committee of the hospital and met the requirements of the Standards of Technical Management for Treatment by Radioactive Particle Implantation (2017 edition) (GUOWEIBANYIFA [2017]). Patients in both groups completed four cycles of NAC by the gemcitabine + cisplatin (GC) regimen before the operation. Finally, among 45 patients in the study group, including 30 (66.7%) males and 15 (33.3%) females, with a median age of 62.7 (range: 46–75) y, 9 (20.0%) presented local lymph node metastasis in the pelvic cavity. A total of 41 (65.9%) patients comprised the control group, including 27 (65.9%) males and 14 (34.1%) females, with a median age of 64.1 (47–75) y; of these 8 (19.5%) had local lymph node metastasis in the pelvic cavity. The baseline clinical data of the patients in the two groups did not differ significantly (all *p* > .05; Table 1).

**Table 1.** Comparison of clinical data between the two groups.

Clinical characteristics	Control group (*n* = 41)	Study group (*n* = 45)	χ^2^	P
Age (years)			0	0.992
≤60	21 (51.22)	23 (51.11)		
>60	20 (48.78)	22 (48.89)		
Tumor diameter (cm)			0.985	0.321
<4	13 (31.71)	10 (22.22)		
≥4	28 (68.29)	35 (77.78)		
Pathology			0.095	0.758
High-grade	33 (80.49)	35 (77.78)		
Low-grade	8 (19.51)	10 (22.22)		
TNM			0.561	0.454
T3	30 (73.17)	36 (80)		
T4	11 (26.83)	9 (20)		
Lymph node metastasis			0.003	0.955
N0	33 (80.49)	36 (80)		
N1-N2	8 (19.51)	9 (20)		

The inclusion criteria were as follows: (1) clinical stage of ≥ cT_3_ or N_1_-N_2_; (2) ECOG score of 0–1 point; (3) without distal metastasis before NAC; (4) underwent RC + pelvic lymph node dissection; (5) signed informed consent. The exclusion criteria were as follows: (1) postoperative pathological findings showed < pT_3_; (2) did not complete preoperative NAC.

Patients in both groups underwent radical cystectomy (RC) + pelvic lymph node dissection. We expected to remove at least 10–12 lymph nodes for accurate staging. However, due to the high proportion of late-stage patients, the difficulty of surgery, and considerations for patient safety and quality of life, on average, only four lymph nodes were targeted during the procedures, and the maximal size of the lymph nodes targeted was 12 cm.

### Preoperative preparations

Radioactive iodine [^125^I] sealed source (referred to as particles) consisted of central silver bars and imported high-purity titanium tubes. The dimensions of the central silver bar were as follows: diameter 0.5 mm, length 4.5 mm, and thickness 0.55 mm. The iodine [^125^I] has a half-life of 59.43 d and a half-value layer of 0.025 mmPb, which generates γ-rays with the initial dose rate of 7 cGy/h capable of permeating 1.7-cm-thick tissues. The radioactive activities of the single particles vary in the range of 0.1–6.0 mCi. The particles used in this study were produced by Seeds Biological Pharmacy (Tianjin) Ltd.

Cranial, thoracic, and abdominal CT scanning assessed the presence of distal metastases. Imaging examinations, such as pelvic magnetic resonance imaging (MRI), were performed to assess peribladder adipose tissue invasion, pelvic lymph node enlargement, and the clinical stages. Patients with the clinical stages of T_3_-T_4_ received three cycles of NAC before preoperative preparations for brachytherapy. The three-dimensional (3D) visual stereoscopic imaging was performed to reconstruct the bladder tumor and suspected pelvic lymph node metastatic tumor and acquire the tumor-relevant information, such as site, size, shape, and correlation with adjacent viscera. During the operation, RC + pelvic lymph node dissection was performed, and the region surrounding the bladder tumor and pelvic lymph nodes was defined as the tumor bed, which could be residual lesions, i.e. sub-region of the tumor, and deemed as the target region of radiotherapy. The matched peripheral dose, i.e. the peripheral dose of the tumor, was selected as the dose for the target region. The radiation dose for UC was 70–75 Gy, the radiation dose for lymph node metastasis was 50–60 Gy, and the targeting dose was approximately 1/5–1/4 of the dose of radiotherapy for tumors. Therefore, the radiation dose was 14–18 Gy for tumor bed of bladder tumor and 10–15 Gy for tumor bed of lymph node metastases. Particles with the radioactive activity of 0.5 mCi were selected for therapy. The interval between two particles in the arrangement was 1 cm, and the equivalent radioactivity at 0.5 cm between two particles was 4285 cGy; at least four to six particles were implanted in the target region. The normal tissues have far higher radiation tolerance doses than the dose of radiotherapy for tumors.

### Surgical methods

Patients in both groups underwent RC + pelvic lymph node dissection. The preoperative preparations in both groups included routine preparations and deciding on the particle implantation plan. During the operation, particle implantation needles were inserted into the tumor bed under the guidance of ultrasound or direct vision, with the interval of 0.5–1.0 cm between the needles and the vertical depth of insertion <1.0 cm; then one particle was implanted at this site. The >2.0-cm implant gun was used to implant two particles with the vertical interval of 1 cm in the backward mode. The particle implantation was performed carefully to avoid injuries to major blood vessels. After the implantation, local compression was performed, followed by hemostasis and fixation of the particles. A self-designed biogel (biological glue) particle blockchain suturing method was used and was employed for the tumor bed on the tissues surrounding the blood vessels, nerves, and the local pelvic wall, which was then wrapped by the surrounding adipose and connective tissues and sutured to fix the particles. The self-designed biogel particle blockchain was as follows: after intraoperative radiation protection was effectuated, one particle was vertically implanted by the implant gun, followed by four to six particles implanted sequentially with the interval of 1 cm on a gelatin sponge. The biogel was applied to the surface of the gelatin sponge, which was wrapped by a hemostatic gauze and sutured for fixation.

### Preoperative NAC

Before NAC, cystoscopy was performed, and the lesion was proven as UC by biopsy and pathological examination. Then, neoadjuvant GC chemotherapy was administered as follows: intravenous injection of gemcitabine 100 mg/m^2^ on d 1 and 8, and intravenous injection of cisplatin 70 mg/m^2^ on d 2. A 3-week treatment was considered one cycle, and all patients were treated for four cycles. Surgery was performed 3–4 weeks after the NAC was completed. Blood routine, urine routine, liver function, renal function, and electrocardiography (ECG) were conducted 1 d before chemotherapy and 1, 8, and 14 d after chemotherapy. Complications and adverse events were detected and treated promptly.

### Follow-up

Plain computed tomography (CT) scanning of the target regions of particle implantation was performed on d 3–5 after the operation in the study group to examine the particle number and shifting. The adverse events were assessed during and 2 weeks after chemotherapy. The complications of particle implantation were assessed 1 week to 1 month as well as 3 months after the operation. The blood and urinary routine, blood biochemistry, ECG, thoracic X-ray, and abdominal color ultrasound were reexamined in both groups, and thoracic and abdominal CT scanning was performed if necessary.

### Efficacy and adverse event assessment

The primary endpoint of this study was the 3-y locoregional recurrence-free survival (LRFS). The secondary endpoints included 3-y distant metastasis-free survival (DMFS), disease-free survival (DFS), and overall survival (OS). The safety endpoint was the incidence of short- and long-term adverse events.

LRFS was defined as the time from treatment completion to the appearance of recurrent lesions in the pelvic cavity (lesions of internal/external iliac lymph nodes, obturator lymph nodes, anterior sacral lymph nodes, cystectomy bed, and lateral pelvic wall) within 30 d after the first discovery of distal metastases. DMFS was defined as the time from treatment completion to the discovery of extrapelvic metastases (including lymph nodes outside the true pelvis and viscera). DFS was defined as the time from treatment completion to local recurrence of tumor, distal metastases, or death due to tumor progression. OS was defined as the time from treatment completion to the death of the patient. Adverse events were assessed by the World Health Organization (WHO) classification criteria. The adverse events after treatment were defined as the adverse events from the start of chemotherapy to 2 weeks after one cycle of completed chemotherapy, from 1 week after the operation to 1 month after particle implantation, and within 3 months after the treatment. The LRFS rate, DMFS rate, DFS rate, OS rate, and short- and long-term incidence of adverse events were compared between the two groups. The adverse events of chemotherapy were assessed according to the WHO criteria for adverse events of chemotherapy (1997 edition).

### Statistical analysis

SPSS 26.0 software was used for statistical analysis. Median (range) and frequency (%) were used to describe the quantitative and qualitative data, respectively. Continuous data were described by median (range) and compared by Mann–Whitney U test. Categorical data were described by frequency (%) and compared using chi-square test. Kaplan–Meier method was used to estimate LRFS, DMFS, DFS, and OS, which were analyzed by log-rank test. Multivariate Cox regression was used to assess the independent predictors of LRFS, DFS, DMFS, and OS. *p* < .05 was considered statistically significant.

## Results

**Figure 1.**
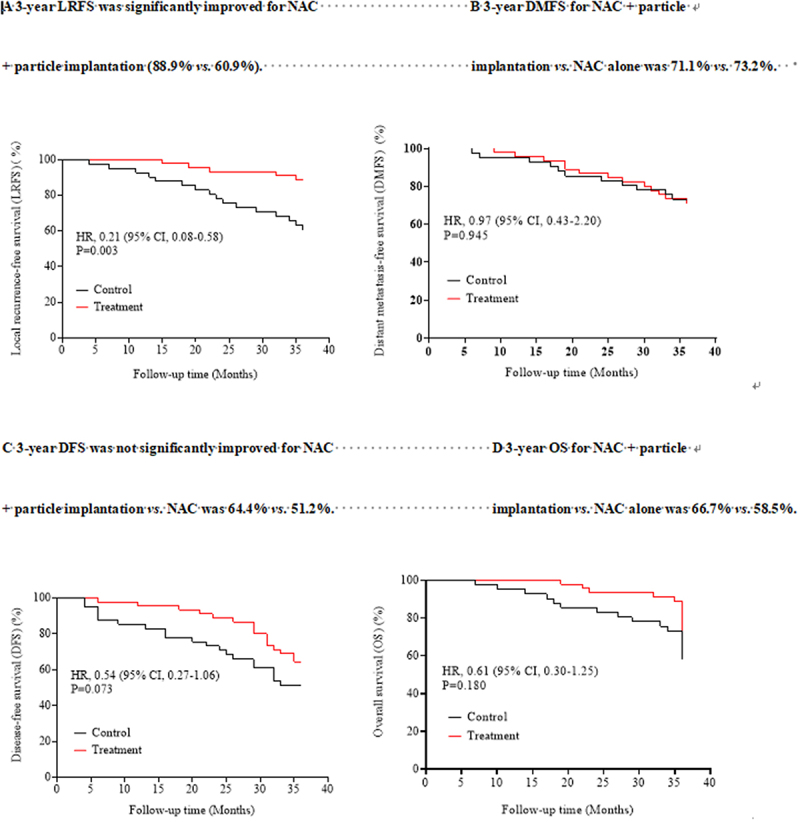
Comparison of Kaplan–Meier survival curves of patients with locally advanced UC in the study group (particle implantation + NAC) *vs*. control group (NAC).

Approximately 30–48 particles were implanted in the patients in the study group, and the mean number of particles was 36. A total of 36 and 9 patients in the study group and 30 and 11 patients in the control group presented stage T_3_ and T_4_ tumors, respectively. In addition, nine and eight patients in the study and control groups exhibited local lymph node metastases, respectively. The median follow-up time of the patients in the two groups was 32.5 (9–42) months. The 3-y LRFS, DMFS, DFS, and OS after the operation were assessed in both groups. The 3-y LRFS was 88.9% (40/45) and 60.9% (25/41) in the study and control groups, respectively, and the difference between the two groups was statistically significant (hazard ratio (HR) 0.210, 95% confidence interval (CI): 0.08–0.58, *p* = .003). The 3-y DMFS was 71.1% (32/45) and 73.2% (30/41) in the study and control groups, respectively, and the difference was not significantly different (HR 0.97, 95% CI: 0.43–2.20, *p* = .945). The 3-y DFS was 64.4% (29/45) and 51.2% (21/41) in the study and control groups, respectively, and the difference between the two groups was not statistically significant (HR 0.54, 95% CI: 0.27–1.06, *p* = .073). The 3-y OS was 66.7% (30/45) and 58.5% (24/41) in the study and control groups, respectively, and the difference was not statistically significant (HR 0.61, 95% CI: 0.30–1.25, *p* = .180). The Kaplan–Meier survival curves of the study and control groups are shown in Figure 1.

The multivariate regression showed that brachytherapy + NAC was a significant predictor of LRFS (HR 0.210, 95% CI: 0.08–0.58, *p* = .003). Other factors, such as age, tumor size, tumor types, pathological stages, and lymph node metastases, could not predict LRFS. Local lymph node metastases are associated with local recurrence. Therefore, although lymph node metastasis was not a statistically significant covariate, it was still included as an adjustment factor and could be a marginal predictor of LRFS (HR 2.54, 95% CI: 0.92–7.00, *p* = .073; Table 2). Multivariate regression analysis did not find any significant predictor of DMFS in this study. The 3-y DMFS was not significantly different between the brachytherapy + NAC group (71.1%; 32/45) and NAC group (73.2%; 30/41) (HR 0.97, 95% CI: 0.43–2.20, *p* = .945). Lymph node metastases were a significant independent predictor of DMFS (HR 2.48, 95% CI: 1.01–6.13, *p* = .048; Table 2). Multivariate regression did not find any significant predictor of DFS in this study. Moreover, the 3-y DFS was not significantly different between the brachytherapy + NAC group (64.4%; 29/45) and NAC group (51.2%; 21/41) (HR 0.54, 95% CI: 0.27–1.06, *p* = .073). However, particle implantation + NAC and lymph node metastases could be marginal predictors of DFS (HR 2.17, 95% CI: 0.97–4.89, *p* = .061; Table 2). Multivariate regression did not find any significant predictor of OS in this study. The 3-y OS was not significantly different between the brachytherapy + NAC group (66.7%; 30/45) and the neoadjuvant chemotherapy group (58.5%; 24/41) (HR 0.61, 95% CI: 0.30–1.25, *p* = .180); however, particle implantation + NAC and lymph node metastases were marginal predictors (HR 2.25, 95% CI: 0.99–5.1, *p* = .052; Table 2).

**Table 2.** Multivariate Cox regression analysis: LRFS, DFS, DMFS, and OS.

Variable	HR	P
LRFS		
Operation	0.210 (0.08–0.58)	0.003
Age	0.49 (0.19–1.23)	0.127
Tumor diameter	0.61 (0.25–1.49)	0.282
Pathology	1.39 (0.5–3.83)	0.527
TNM	1.09 (0.38–3.12)	0.867
Lymph node metastases	2.54 (0.92–7.00)	0.073
DFS		
Operation	0.54 (0.27–1.06)	0.073
Age	0.67 (0.34–1.31)	0.239
Tumor diameter	0.74 (0.36–1.5)	0.402
Pathology	0.99 (0.43–2.26)	0.972
TNM	1.15 (0.53–2.47)	0.730
Lymph node metastases	2.17 (0.97–4.89)	0.061
DMFS		
Operation	0.97 (0.43–2.20)	0.945
Age	0.73 (0.32–1.65)	0.442
Tumor diameter	0.56 (0.24–1.3)	0.176
Pathology	1.53 (0.61–3.81)	0.363
TNM	1.32 (0.52–3.4)	0.56
Lymph node metastases	2.48 (1.01–6.13)	0.048
OS		
Operation	0.61 (0.30–1.25)	0.180
Age	0.69 (0.34–1.41)	0.309
Tumor diameter	0.63 (0.3–1.32)	0.220
Pathology	1.08 (0.45–2.59)	0.856
TNM	1.28 (0.59–2.81)	0.532
Lymph node metastases	2.25 (0.99–5.1)	0.052

Assessment of adverse events: Local particle shifting was detected in three patients in the study group at 1 week to 1 month after the operation (criteria of local particle shifting: findings of abdominal and pelvic plain CT scanning 1 d after operation were compared to those of abdominal and pelvic plain CT scanning at 1 week to 1 month after the operation), while no related complications occurred. Also, no intestinal canal or blood vessel damage was detected, but other complications of particle implantation, such as no poor incision union, were observed. The incidence of particle implantation-related adverse events, such as bearing down sensation, was significantly higher in the study group than in the control group (p < .01); most of these events were grade 1–2. Patients in both groups completed the GC chemotherapy, and the adverse events were assessed. Blood events (anemia, leukocytopenia, and thrombocytopenia), liver and renal dysfunction, vomiting, diarrhea, and weakness were the major adverse reactions, which were alleviated after symptomatic treatments. The results of the incidence of adverse events in the two groups are shown in Table 3.

**Table 3.** Comparison of adverse events in UC patients between the study and control groups [n (%)].

Adverse event	Study group (n = 45)		Control group (n = 41)		*P* value
	Grade 1-2	Grade 3-4	Grade 1-2	Grade 3-4	
Anemia	24 (53.3)	13 (28.9)	20 (48.8)	5 (12.2)	0.532
Leukocytopenia	35 (77.8)	4 (8.8)	30 (73.1)	3 (7.3)	0.132
Thrombocytopenia	36 (80.0)	6 (13.3)	30 (73.3)	5 (12.2)	0.346
Liver dysfunction	11 (24.4)	0 (0)	14 (29.3)	0 (0)	0.165
Renal dysfunction	16 (35.5)	2 (4.4)	12 (29.2)	0 (0)	0.361
Malignant vomit	28 (62.2)	17 (37.8)	22 (53.7)	14 (34.1)	0.532
Diarrhea	21 (46.7)	7 (15.6)	8 (19.5)	3 (7.3)	0.062
Weakness	31 (68.9)	5 (11.1)	20 (48.8)	1 (2.4)	0.072
Bearing down sensation	2 (4.4)	0 (0)	0 (0)	0.002	15 (33.3)

## Discussion

Pathological stage is the major influencing factor of the prognosis of UBC patients after RC; the 5-y OS is <40% for patients with locally advanced UBC (T_3_-T_4_)^8^ and is about 30% for patients with positive lymph nodes.^20^ How to improve the postoperative survival of patients with locally advanced UBC (T_3_-T_4_) has been a clinical research hotspot. Several studies have demonstrated that Platinum-based combination NAC has a high response rate for bladder cancer. Platinum-based NAC in combination with RC + pelvic lymph node dissection is the standard treatment for MBIC in clinical practice.^21–24^ NAC improves the survival by about 5–10% and reduces the risk of death by 16–33% compared to patients who underwent only RC.^24–28^ In this study, all 86 patients with stage T_3_-T_4_ UBC underwent NAC in combination with RC + pelvic lymph node dissection.

Local recurrence after operation for pT_3_-T_4_ UBC significantly influences the treatment effects, which is associated with the low cancer-specific survival.^12,13^ For such patients, the effects of salvage treatment are poor,^9,11–14^ and the median survival was only about 9 months.^9,15^ Various retrospective studies on surgical treatments have demonstrated^29^ that although no lymph node metastases were detected by postoperative pathological examinations, extensive lymph node dissection could improve the survival rate. These findings indicated that preoperative NAC + intraoperative radioactive particle implantation could clear the hidden lymph node micro-metastases to reduce the local recurrence and distal metastases and consequently improve the survival of patients. The findings of clinical studies have demonstrated that NAC reduces the risk of local recurrence. Compared to adjuvant chemotherapy alone, radical cystectomy + chemoradiotherapy significantly improves the DFS and might prolong the OS.^16–18,30^ Zaghloul et al.^31^ provided adjuvant chemoradiotherapy for patients with locally advanced bladder cancer who underwent RC and compared the findings with patients who received only adjuvant chemotherapy. The results showed that patients had a high tolerance to adjuvant chemotherapy + radiotherapy, which significantly improved the LRFS and prolonged the DFS compared to patients who received only chemotherapy.

Since vital viscera, such as the small intestine in the abdominal and pelvic cavities, have a low tolerance to routine external radiotherapy, it is not recommended to apply the high dose external radiotherapy to control severe recurrent bladder cancer as the treatment is in close proximity to such viscera.^32^ Although the adjuvant local treatment did not improve the survival, it prevented the local recurrence in the pelvis; the recurrence induces local pain, edema of lower limbs due to compression on veins and lymph vessels, and uronephrosis due to ureteral obstruction.^15,33,34^

Compared to conventional external radiotherapy, brachytherapy by implantation of radioactive particles has the following advantages: (1) broader indications and shorter hospital stay; (2) tumor tissues are exposed to low-dose radioactive rays for a prolonged period, which leads to tumor cell apoptosis, while the effects on the cells of normal tissues are low. Therefore, interstitial implantation of radioactive particles for the treatment of malignant tumors has definite treatment effects and low adverse effects and thus has been extensively applied in clinical practice.^35,36^ Several studies have demonstrated the efficacy of brachytherapy in the treatment of urothelial carcinoma. For instance, a study by Zaghloul et al. highlighted the improved locoregional control in muscle-invasive bladder cancer patients who received brachytherapy. Similarly, Cozzarini et al. reported enhanced disease-free survival (DFS) with the use of brachytherapy in combination with radical cystectomy (RC) and neoadjuvant chemotherapy (NAC). These studies underscore the potential of brachytherapy to reduce local recurrence and improve overall survival in patients with bladder cancer.

In addition to its application in bladder cancer, there is growing interest in the use of brachytherapy for upper tract urothelial cancers (UTUC). Xuebing Han et al. conducted a series of studies on the interstitial implantation of radioactive particles for the treatment of UTUC. Their findings suggested that brachytherapy, combined with surgery and chemotherapy, was safe and effective, resulting in lower adverse events and potential overall survival benefits. This indicates that the principles and advantages of brachytherapy in bladder cancer could be translated to the treatment of UTUC. We emphasize on further research and clinical trials to fully establish the efficacy and safety of brachytherapy in the treatment of upper tract urothelial cancers. Continued discoveries in this field can provide additional treatment options for UTUC patients, potentially improving their prognosis and quality of life.

Furthermore, the use of brachytherapy in UTUC offers several advantages over conventional external radiotherapy. The precise delivery of radiation to the tumor site minimizes damage to surrounding healthy tissues, which is particularly important in the anatomically complex regions of the upper urinary tract. The prolonged low-dose exposure characteristic of brachytherapy leads to effective tumor cell apoptosis with fewer side effects.

Given the success observed in bladder cancer and the initial positive outcomes in UTUC, further research and clinical trials are warranted to fully establish the efficacy and safety of brachytherapy in the treatment of upper tract urothelial cancers. Continued exploration in this area could provide additional therapeutic options for patients with UTUC, potentially improving their prognoses and quality of life.

To date, only a few studies have investigated the radiotherapy of locally advanced UC, and the treatment efficacy was not definite. Herein, the clinical observations of 22 patients with locally advanced UC who received treatment of particle implantation and presented good treatment efficacy have been reported.^37^ The findings of this series of studies^19^ showed that implantation of radioactive ^125^I particles in combination with surgery + chemotherapy for the treatment of locally advanced (stage T_3_-T_4_) UTUC has lower adverse events than the treatment with surgery + chemotherapy; also, it was safe and effective and could achieve OS benefits.

A high incidence of local micro-metastases is recorded in patients with locally advanced (T_3_-T_4_) UBC,^38^ and thus LRFS was selected as the primary endpoint in this study. The findings in this study showed that additional intraoperative particle implantation for brachytherapy could significantly improves the LRFS, and the absolute improvement of 3-y LRFS could be about 28% (88.9% *vs*. 60.9%), and the difference between the study and control groups was statistically significant (HR 0.210, 95% CI: 0.08–0.58, *p* = .003).

The 3-y DFS was 64.4% (29/45) and 51.2% (21/41) in the two groups, respectively, and the difference was not statistically significant. However, after additional intraoperative particle implantation for brachytherapy was performed, the substantial benefits of the 3-y LRFS converted to the marginal benefits of the 3-y DFS (64.4% *vs*. 51.2%, *p* = .073). The 3-y OS was 66.7% (30/45) and 58.5% (24/41) in the two groups, respectively, and the difference was not statistically significant between the two groups.

In this study, examination at 1 week to 1 month after operation in the surgery group showed local particle shifting in three patients, while no related complications were observed. In addition, no damage to the intestinal canal or blood vessels, poor union of incision, or other complications of particle implantation was found. The incidence of particle implantation-related adverse events, such as bearing down sensation, was significantly higher in the study group than in the control group at 3 months after the operation (*p* < .01); most of these events were grade 1–2. Therefore, the incidence of complications of particle implantation was similar to that reported previously and significantly lower than the adverse events of conventional external radiotherapy.^31,32^

Nevertheless, the present study had some limitations. The sample size of this study was small, and the follow-up time was short. Thus, additional studies with more patients and longer observation times are needed to improve the significance of the series study of interstitial implantation of radioactive particles. Addtionally, the retrospective design inherently carries potential biases, such as selection bias and information bias. Retrospective studies rely on existing records, which may not capture all relevant data consistently. Thirdly, this study was conducted at a single center, which may limit the generalizability of our findings. The patient population and treatment protocols at our institution might differ from those at other centers, potentially affecting the external validity of the results.

## Conclusions

In summary, compared to the neoadjuvant chemotherapy alone, intraoperative implantation of radioactive particles + the neoadjuvant chemotherapy for the treatment of patients with locally advanced urothelial bladder carcinoma who underwent radical cystectomy + pelvic lymph node dissection significantly improves the locoregional recurrence-free survival and prolongs the disease-free survival, thus acquiring survival benefits. In addition, the treatment was safe and effective, with only limited adverse events.

## Data Availability

The authors confirm that the data supporting the findings of this study are available within the article.

## Abbreviations

UBC: Urothelial Bladder Carcinoma
NAC: Neoadjuvant Chemotherapy
RC: Radical Cystectomy
LRFS: Locoregional Recurrence-Free Survival
DMFS: Distant Metastasis-Free Survival
DFS: Disease-Free Survival
OS: Overall Survival
CT: Computed Tomography
MRI: Magnetic Resonance Imaging
ECOG: Eastern Cooperative Oncology Group
